# Practical Guidance and Ethical Considerations for Studies Using Photo-Elicitation Interviews

**DOI:** 10.5888/pcd11.140216

**Published:** 2014-10-30

**Authors:** Eva Bugos, Rosemary Frasso, Elizabeth FitzGerald, Gala True, Anna M. Adachi-Mejia, Carolyn Cannuscio

**Affiliations:** Author Affiliations: Eva Bugos, Elizabeth FitzGerald, Perelman School of Medicine at the University of Pennsylvania, Philadelphia, Pennsylvania; Rosemary Frasso, Perelman School of Medicine, Center for Public Health Initiatives, and School of Nursing, University of Pennsylvania, Philadelphia, Pennsylvania; Gala True, Center for Health Equity Research and Promotion, Philadelphia Veterans Affairs Medical Center, and Perelman School of Medicine at the University of Pennsylvania, Philadelphia, Pennsylvania; Anna M. Adachi-Mejia, Geisel School of Medicine at Dartmouth, Norris Cotton Cancer Center, and Dartmouth Institute for Health Policy and Clinical Practice, Lebanon, New Hampshire. Dr Cannuscio is also affiliated with the Center for Public Health Initiatives at the University of Pennsylvania, Philadelphia, Pennsylvania.

## Abstract

Photo-elicitation is a qualitative interviewing technique that has gained popularity in recent years. It is the foundation for photovoice projects and is a tool well-suited for community-based participatory research. Photo-elicitation yields rich data, and interview participants say these interviews encourage community awareness and engagement. This article draws on 9 studies, conducted by researchers at 3 institutions (the University of Pennsylvania, the Philadelphia Veterans Affairs Medical Center, and the Geisel School of Medicine at Dartmouth) in partnership with community-based organizations and students, in which 303 participants completed photo-elicitation interviews. We offer 8 practical suggestions for overcoming challenges encountered during photo-elicitation research and for managing ethical concerns about the use of visual data in public health research. Our guidelines can inform study design, protocol development, and institutional review board approval.

## Introduction

Photo-elicitation is a qualitative research technique developed in 1957 that uses images to prompt and guide in-depth interviews (1). Researchers ask members of the community under study to photograph or videotape their environment. The photographers then comment on the images they take. Photo-elicitation is a core component of photovoice, which was first described in 1997 and is a form of community-based participatory research that engages participants at each step of the research process as documentarians, commentators, and agents of social and political change (2,3). The terms “photo-elicitation” and “photovoice” are often used interchangeably. However, we make the distinction that photo-elicitation focuses on the interview process itself, whereas photovoice is a more comprehensive term reflecting an action-oriented research strategy. A Web of Science search in 2000 for studies that included the term “photovoice” produced only 5 articles; by 2013, the cumulative number of articles had grown to 359 (Figure), the increase driven in part by the use of digital cameras and smartphones.

**Figure:**
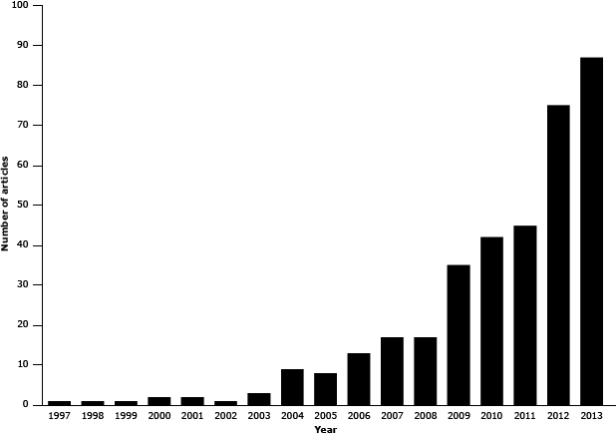
Number of Web of Science articles found for the search term “photovoice” as topic each year from 1997 through 2013. **Table:** 

Year	No. of Articles
	
1997	1
1998	1
1999	1
2000	2
2001	2
2002	1
2003	3
2004	9
2005	8
2006	13
2007	17
2008	17
2009	35
2010	42
2011	45
2012	75
2013	87

The increasing use of photovoice has created growth in the use of photo-elicitation, especially among vulnerable populations such as homeless and low-income youths, senior citizens, cancer survivors, and indigenous people in the United States and Canada (2,3). Despite this increase, few publications detail the challenges of conducting photo-elicitation interviews either as a process by itself or in the context of photovoice studies. This information gap may delay the progress of studies using the photo-elicitation technique because instead of relying on guidance in the scientific literature, researchers must learn about the technique through trial and error. 

We analyzed a convenience sample of 9 studies (4–12) conducted by the authors at 3 institutions (the University of Pennsylvania, the Philadelphia Veterans Affairs Medical Center, and the Geisel School of Medicine at Dartmouth) in partnership with community-based organizations and students, in which 303 participants completed photo-elicitation interviews (Table 1). Our objective was to synthesize the information in the studies to offer guidance on the practical and ethical considerations of photo-elicitation. The 9 studies include a range of research using the photo-elicitation technique, whether used by itself or in a photovoice study. We describe challenges encountered in conducting photo-elicitation interviews and suggest practical solutions, including strategies for collecting and managing photographs, choosing an interview location, developing the interview guide, and troubleshooting the interview. We also describe our approaches to anticipating and managing ethical concerns on the use of visual data in public health research, including photography ethics training and protecting participant confidentiality and privacy. Our guidance can inform study design, protocol development, and institutional review board (IRB) approval. Although this article focuses on the details of photo-elicitation, we remind readers that photo-elicitation interviews must also adhere to best practices for in-depth interviews and qualitative research in general (13).

**Table 1:** Sample of 9 Photo-Elicitation Studies Approved by Institutional Review Boards at 3 Institutions^a^

Study	Photo-Elicitation	Photovoice	Mission	Participants
A Place to Call Home (4)	**√**	**√**	Engage and empower young adults in Philadelphia to understand how home, as they define it, contributes to or takes away from their sense of well-being. Methods included photography, storytelling, and art.	48 young adults experiencing housing insecurity and homelessness
journey2home (5)	**√**	**√**	Use public art to illuminate the challenges of housing insecurity for young adults and engage the Philadelphia community in hearing their stories.	25 young adults, many experiencing housing insecurity and homelessness
Health of Philadelphia Photo-documentation Project (6)	**√**		Assess Philadelphia residents’ perspectives on the causes and consequences of urban health disparities.	32 adults from neighborhoods with distinct socioeconomic characteristics
Youth Vision (7)	**√**		Understand views among adults and young adults on challenges and opportunities for change in the urban food, physical activity, and tobacco environments.	48 adults and young adults
Exploring disparities in an urban setting: research students turned researchers (8)	**√**		Engage students as researchers to explore how other students on an urban campus experience and define disparity	17 graduate students each enrolled 1 participant
Learning by doing: students explore the meaning of safety on an urban campus (9)	**√**		Engage students as researchers to explore how other students on an urban campus experience and define safety	21 graduate students each enrolled 1 participant
Under pressure: students explore stress on an urban campus (10)	**√**		Engage students as researchers to explore how other students on an urban campus experience and manage pressure	14 graduate students each enrolled 1 participant
From War to Home: Photovoice as an Educational Intervention to Improve Care of OEF/OIF Veterans (11)	**√**	**√**	Explore combat veterans’ perspectives on the impact of military service and deployment on health, challenges to making the transition home, and barriers to receiving care for war-related injuries.	40 US veterans of the wars in Iraq (Operation Iraqi Freedom) and Afghanistan (Operation Enduring Freedom)
Fruits, Vegetables, Activity, and Sleep: A Dartmouth Photovoice Study (12)	**√**	**√**	Elicit perspectives on barriers and facilitators to eating fruits and vegetables, being active, and getting enough sleep.	52 middle and high school students, 6 senior adults in rural New Hampshire

Abbreviation: OEF/OIF, Operation Enduring Freedom/Operation Iraqi Freedom.

a The sample was a convenience sample of photo-elicitation studies conducted by the authors at 3 institutions: University of Pennsylvania, the Philadelphia Veterans Affairs Medical Center, and Dartmouth College (4–12).

## Guidelines for Conducting Photo-Elicitation Interviews

### 1. Build a strong foundation for interviews by requiring ethical photography practices.

Photographs can leave an indelible impression, generate public conversation, and even ignite social change by bridging disparate social worlds and offering glimpses of what might otherwise remain unseen. Given the power of photography, project teams intending to conduct photo-elicitation interviews should engage team members in discussions about ethically sound photography practices, such as how and when to ask for photograph releases. For example, in the Health of Philadelphia Photo-documentation Project (HOPPP), investigators sought to understand the health priorities and beliefs of adult urban residents on the underlying causes of good or poor health (6). During individual and group training sessions, HOPPP researchers demonstrated the basics of good photography, discussed the ethical challenges of taking photographs with community participants, and presented guidelines for overcoming the challenges (Table 2). These guidelines are not intended as legal advice; rather, they are practical strategies developed by the project team and the University of Pennsylvania IRB. They may not be appropriate for other studies; researchers should work with their own project teams and IRBs to tailor their own guidelines. For example, some researchers may opt to require a written photograph release from any identifiable individual in a photograph, while others may ask team members not to photograph people at all. Wang and Redwood-Jones offer a thorough discussion of image ethics and privacy issues for photovoice projects (14).

**Table 2:** Ethical Challenges for Participant Photographers and Project Strategies Approved by University of Pennsylvania Institutional Review Board for the Health of Philadelphia Photo-Documentation Project

Challenge	Strategy
When should I seek verbal permission or a photo release?	Although various studies have used different approaches, in this study, verbal permission was obtained before photographing individuals in groups of 4 or fewer people when they were photographed at close range and were therefore the main subject of the photo (eg, on the same side of the street or within a distance that allowed people to converse at a normal conversational volume).
When do I *not* have to obtain verbal permission or a photo release?	Verbal permission was not required for photographs taken of groups of 5 or more individuals in public places, because no person was the subject of the photograph. Additionally, if an individual was not the main focus of the photograph but was incidental to it (eg, a shopper was walking out of a grocery store across the street from where the photographer was standing) or if the individual was not identifiable in a photograph (eg, the photo was taken from behind or from a distance), permission or a release was not required. Other studies, especially photovoice, use a different approach; Wang and Redwood-Jones required a written release for any photographs that included people (14). Journals often describe their own requirements for obtaining consent from identifiable subjects. For example, the *AMA Manual of Style, 10th Edition*, specifies that “the author should obtain and submit a signed statement of informed consent” from people who are identifiable in photographs or videos to be published (15).
How can I obtain permission or a photo release?	To obtain verbal permission, participants introduced themselves, explained the purpose of the photography, and asked for permission to take a photograph.
What should I do about permission or photo releases in a private location, like a home?	Participants obtained verbal permission to photograph people in private settings.
Who do I talk to for permission or photo releases to photograph minors?	Participants sought the permission of a parent or guardian before taking photographs of minors.
What do I do if anyone asks me not to take a photograph?	If anyone asked participants not to take a photograph, the request was to be honored, even if in a public place. Additionally, if the photographer sensed any “reluctance, confusion, or disdain,” they were instructed to refrain from taking the photograph. Above all, participants were instructed to “respect a person’s right to refuse to be photographed” (14). UNICEF provides helpful guidelines on photographing or videotaping children (16).

Researchers should review the ethical concerns unique to each photo-elicitation project setting and population. For example, several projects in our sample (4–6) took place in disadvantaged and high-crime urban neighborhoods. The research teams emphasized that the safety of photographers and photograph subjects trumped all other concerns. In journey2home (5) and A Place to Call Home (4), photovoice projects that focused on housing insecurity among young adults, the project teams coached the participant-photographers (all of whom were young adults) to relinquish cameras immediately if someone wanted to steal the equipment. To the surprise of the research team, the participants protested having to give up their cameras. Therefore, the team’s discussion about safety was unexpectedly extended during multiple sessions to generate troubleshooting strategies and build self-efficacy among participants for maintaining safe conduct.

During several other projects, participant-photographers were encouraged to team up with partners when they took photographs in the community. In the Youth Vision project (7), which explored the perspectives of adults and young adults on the food and tobacco environments in Philadelphia, researchers told participant-photographers not to photograph illegal activity or people who indicated (verbally or nonverbally) that they did not want to be photographed (7,17). Researchers should instruct participants at every meeting not to take photographs that indict, stigmatize, embarrass, or shame individuals or groups (14). Participants should be instructed to take photographs during their normal daily routines, in familiar public places, near work and school, and at home. Table 3 offers a starting point for anticipating potential ethical dilemmas that may arise during a photo-elicitation project by reviewing challenges and resolutions unique to each study in our sample.

**Table 3:** Challenges and Solutions in a Convenience Sample of 9 Photo-Elicitation Studies Approved by Institutional Review Boards^a^

Study title	Challenge	Resolution
A Place to Call Home (4)	Youth and young adult participants were not expected by some team members and community partners to sustain participation in the program, because of challenging life circumstances, including poverty and housing insecurity.	The project team offered assistance with transportation, provided food, and mentored students through group photography outings in urban neighborhoods. Interviews were conducted on designated interview days, during which 4 or 5 interviewers worked simultaneously to connect with as many students as possible.
journey2home (5)	Young adult participants were resistant to using digital equipment that was viewed as “uncool.”	The project team sought participants’ views on preferred digital equipment. In later project phases, an effort was made to give all participants the same preferred digital equipment.
Health of Philadelphia Photo-documentation Project (HOPP) (6)	The research team used both staff- and participant-generated photographs during photo-elicitation interviews. Participants did not seem highly motivated to discuss staff-generated photographs. However, discussion of participants’ own photographs yielded detailed, insightful analyses of their concerns and interests.	Researchers moved to focus interviews exclusively on participants’ own photographs as HOPPP progressed. In addition, the researchers’ subsequent project protocols only called for participant-generated photographs to be used during photo-elicitation interviews.
Youth Vision (7)	Youth participants’ photographs focused disproportionately on food and nutrition relative to tobacco, despite the fact that participants were asked to focus on both subjects equally. Relatedly, many participants appeared reluctant to bring up tobacco-related issues, perhaps because of stigma associated with smoking.	What was initially a challenge became a central study finding. Researchers recognized that extensive photography and commentary on nutrition reflected a heightened awareness of food and nutrition issues among youth participants. Tobacco use and prevention were not salient issues for youth and needed to be revived as issues of public concern.
Student Projects (Disparities; Safety; Stress) (8–10)	The first version of the student project gave participants 2 weeks to take photographs and complete an interview. A few participants were unable to complete the interview within that time frame, leaving the student researchers without an interview subject. There was not enough time to identify and train a new participant.	The project director adapted the time line to better fit a semester-long course. The students trained participants at an earlier date so that if a participant was unable to complete the project, the student would have time to identify a replacement. In the most recent course, participants had 1 week to take photographs and complete the interviews. This made participation a shorter-term commitment.
From War to Home (11)	Participants talked about and shared photographs of sensitive subject matter (eg, photographs depicting marijuana use, discussion of purchasing opioids). Others shared photographs taken during a deployment that were possibly illegal and/or had potential to contribute to stigmatization of veterans (eg, photographs of Iraqi prisoners-of-war, aftermath of an explosion that included blood).	The project director told veterans at the time of informed consent that their photographs might be excluded from dissemination if they had potential to harm an individual or veterans as a community. Throughout the project she worked closely with each veteran and the project’s advisory board to determine whether or not to use sensitive photographs in the dissemination phase or project exhibits and when to credit particular photographs to “anonymous.”
Dartmouth Photovoice Study (12)	Participants needed to attend at least 2 meetings (1 training, 1 group interview). Many participants, especially students, were unable to attend a scheduled meeting because of conflicts (eg, illness, team sports conflict, doctor appointments).	The researcher built participant scheduling conflicts into her own expectations and scheduled participants for 4 meetings and a fifth back-up meeting. Participants were invited to attend all meetings. However, even those who only attended only 2 meetings could participate in the study. If a participant missed the photography training session, the researcher trained that participant individually. That participant had to catch up to his peers by taking photographs before attending another meeting.

a The sample was a convenience sample of photo-elicitation studies conducted by the authors at 3 institutions: University of Pennsylvania, the Philadelphia Veterans Affairs Medical Center, and Dartmouth College (4–12).

Some participant-photographers may wish to use photographs taken in the past instead of taking new photographs. In From War to Home (11) which aimed to understand the perspectives of combat veterans on health during deployment and as they made the transition home, participants used photographs they took during deployment — before they were participant-photographers in the photovoice project.

The Dartmouth Photovoice Study (12), which focused on barriers and facilitators to eating fruits and vegetables, being active, and getting enough sleep, managed ethical concerns by asking participants not to photograph people. In the photography training sessions, the researcher asked participants to identify photographs that they liked or disliked in books and magazines. The researcher used these photographs to facilitate a group discussion about general photography concepts like lighting and composition and about ways to convey a concept without photographing a person (eg, using shadows or clothing). The researcher also gave participants a blank logbook for notes about where and why they took each photograph and asked participants to brainstorm photography ideas during the training (5). This strategy may be appropriate for research with particularly vulnerable populations, such as children, or on sensitive topics, such as serious health conditions.

### 2. Develop a consistent photo collection process that aligns with study goals.

Among the 9 photo-elicitation studies in our sample, photographs were taken on disposable film cameras, digital cameras, and smartphones. For simplicity, we recommend that researchers use only one type of device for each study and that they consult participants about the preferred medium. HOPPP participants used film cameras, which limited the number of photographs each participant took and added the step of printing photographs before the interviews. During interviews with HOPPP participants, researchers used a combination of participants’ own photographs and those taken by project staff. Participants discussed their own photographs at length, and discussion was richer and more detailed when they talked about their own photographs than when they talked about staff photographs. On the basis of that experience, researchers decided to use only participant-generated photographs in later studies.

In 3 studies conducted with graduate students at the University of Pennsylvania to understand student perspectives on disparities, safety, and stress, many participants used their smartphones. They e-mailed images to the interviewers, who displayed them on a laptop during the interview. However, relying on e-mail to share photographs during the interview meant that researchers had to relocate or reschedule the interview if the Internet connection failed. Still other studies used digital cameras, and the interviewer loaded pictures onto a laptop or tablet before the interview. Digital cameras allow participants to take many photographs, thus improving the likelihood of having high-quality photographs for use in presentations and exhibits. However, participants who elected to take a large number of photographs had to pre-screen their images and select a manageable set (approximately 10) for interviews.

### 3. Choose an interview location conducive to photo viewing, safety, and privacy.

If researchers decide to use digital images during the photo-elicitation interviews, we advise them to find an interview location that is conducive to projecting images, whether on a computer, via a projector, or on a handheld device. For group interviews, one group of researchers found that a tablet worked better than a laptop for viewing photographs because it was easier to pass among participants (12). When conducting one-on-one interviews, telephones or other handheld electronic devices may be sufficient, but the team should consider the level of photographic detail sought. If the photographs are being used to understand a complex process, then the projection device should provide images of sufficient quality to view details. If the images are primarily used to evoke narratives or metaphors, then detailed viewing may be less critical.

For all interviews, location is key to creating an environment where interviewees feel safe (18). When interviews involve sensitive topics, the interview should be in a private location, such as the researcher’s office or a community site with safe and easy access (for example, a public library or a community center). In journey2home, the team leased a storefront with good access to foot traffic and public transportation (5). The site served both as an interview location and as a venue for social gatherings and displays of participants’ photographs and related artwork and performances (7). In the Dartmouth Photovoice Study, the researcher conducted group interviews with students in a classroom after school. This venue afforded a sense of privacy for students, because the building was quiet and uncrowded and was also convenient and familiar (12). When choosing an interview location, researchers should balance considerations for privacy, convenience, and safety.

### 4. Systematically sort and label all participant photographs.

Researchers should have participants sort and label photographs at the start of the photo-elicitation interview. The process encourages participants to organize their thoughts and allows them to begin the interview by talking about issues they deem most important when they have the most energy. In addition, sorting and labeling re-familiarizes participants with the photographs, which they might have taken several weeks before the interviews.

The method of sorting photographs should align with study goals. For example, at the start of the HOPPP interviews, the participants’ first task was to sort their photographs into 3 piles — one pile each for images of positive, negative, or neutral conditions for health. Within each pile, participants then ranked the photographs, beginning with the most important or best representation of health priorities or concerns in their neighborhood. Those photographs were numbered according to their rank, and participants were instructed to refer to each photograph by its number during the interview, so that text from the interview transcript could be associated with its corresponding photograph. Other strategies may be to group photographs on the basis of whether they represent barriers or facilitators or to organize photographs by theme if the study aims to elicit perspectives on multiple topics.

### 5. Create a photo-elicitation interview guide.

The interview guide should originate from a clearly articulated project mission, which should be developed and refined by the team before project initiation. Interviewees should be conversant in the mission of the project, so that their photographs and associated stories will clearly relate to the mission.

As the defining feature of photo-elicitation, the reliance on photographs provides a shared focus for the interviewer and interviewee. In the hands of an effective interviewer, the guide can help the interviewee reflect on the meaning and content of the photographs, encouraging him or her to draw connections and recall details and interpretations that had not previously been considered. The photo-elicitation interview guide should offer a core set of prompts that address each image directly and encourage storytelling.

Many projects, our own included, use or adapt questions suggested by Wang and Pies (19,20). HOPPP’s interview guide asked an introductory question about each photograph: “Tell me the story of this photograph.” Interviewers followed up with appropriate questions from the following list: “What do you see in this photo? What else is happening here? How does this relate to your life and life within your community? Why does this problem, concern, or strength exist? What can we (or others) do about it? What would you hope for this scene to look like in years to come?”(6).

At the end of the interview, the interviewer should take time to review the interview guide, asking any questions that were overlooked. In addition, researchers should close the interview with a summary question, asking if the participant has anything he or she would like to add about the images or issues discussed. Another approach is to have the interviewee assemble his or her photographs (if in print form) on a table or presentation board and ask, “Looking at all these photographs together, what story do they tell about [the mission of this project]?”

### 6. Be prepared to respond flexibly in challenging interviews.

In-depth interviews can be derailed when a participant goes off-topic, says very little, or says a great deal about one topic to the exclusion of others. We have found that photo-elicitation interviews often flow more smoothly than traditional in-depth interviews, because photographs offer an opportunity to guide the interview to useful terrain. For interviewees who expound on a single topic, interviewers can redirect the participant in a positive, respectful way by asking a question about another photograph. For more reticent participants, photographs deflect attention from the interviewee and toward the photograph, allowing the interviewee to relax. Interviewers may also offer the option of written reflection.

Photo-elicitation interviewers may face challenges if the interviewee brings low-quality images. Interviewers should not skip over these images during the interview, because even a poor-quality photograph can elicit a meaningful and useful response. Instead, the interviewer should simply ask what the interviewee hoped to depict and guide the conversation from there. If the subject matter does not seem immediately relevant, the interviewer should probe for information about the participant’s motivation for the photograph and what meaning the subject matter holds.

### 7. Protect confidentiality and privacy.

Participant-generated photographs pose special challenges to confidentiality and privacy, because images have the potential to visually identify study participants, especially if researchers use images in the dissemination phase of the project. Participants must be told in advance how their photographs may be used. Until the moment of publication, the participant should be given permission to ask that their images not be used, shared, or distributed. If participants request it, photographs should be destroyed, deleted, or returned to participants according to an established IRB-approved protocol.

Member checking, an ongoing process in which researchers ask participants for their input on data analysis and interpretation, is another strategy to respect participant privacy and to establish credibility for qualitative data (21). We have found member checking to be especially important in projects conducted among vulnerable populations, so participants can redact photographs or comments they prefer not to share. That said, in our projects to date, including projects on potentially sensitive subjects like housing insecurity and war-related trauma, few participants have requested that their photographs not be published or displayed publicly.

### 8. Consider the limitations of the photo-elicitation interview technique.

Photo-elicitation has several limitations. The use of visual methods may lead participants to focus on visible, observable phenomena instead of abstract concepts. Another challenge we encountered, albeit rarely, is that younger participants sometimes take more self-portraits or “selfies” than photographs of their environment. However, these challenges are easily overcome by helping participants identify alternate photograph opportunities that have subjects of symbolic significance. Finally, researchers should develop realistic expectations for the technical and artistic strength of participants’ photographs. If the study aims to publish or exhibit photographs, as is often the case for photovoice studies, researchers may need to provide multiple training sessions on photographic technique. Alternatively, researchers in photo-elicitation studies may choose to use a combination of professional and participant photographs in the publication stage.

## Conclusion

This article demonstrates and clarifies concepts from 9 studies approved by the IRBs at the University of Pennsylvania, the Philadelphia VA Medical Center, or Dartmouth College. For researchers interested in undertaking a photo-elicitation study, this article previews the practical and ethical considerations that may arise before the interview (when training participants, choosing an interview location, and creating an interview guide) during the interview (sorting photographs and troubleshooting), and after the interview (protecting confidentiality and privacy).

As photo-elicitation becomes more popular among public health researchers, it is important for practitioners to establish a consensus on best practices for using visual methods in health research. There is ample opportunity to develop and test standardized protocols for simultaneous analysis of photographs and text derived from photo-elicitation studies. In addition, the field would benefit from a thorough examination of participants’ perspectives on the technique itself.

Photo-elicitation is a powerful research technique for connecting with and empowering interviewees, especially when used in conjunction with community-based participatory research approaches such as photovoice. Researchers interested in implementing the technique should anticipate ethical and confidentiality issues and create protocols to deal with practical concerns before undertaking their own studies or writing IRB protocols.
